# Association of *IL4 rs2070874, FoxP3 rs3761548* Polymorphisms with Keratoconus in Algeria

**DOI:** 10.18502/jovr.v16i4.9745

**Published:** 2021-10-25

**Authors:** Wafaa Meteoukki, Mostefa Fodil, Nawel Adda Negaz, Nesrine Rahmoun, Sarah Lardjam Hetraf, Hadjira Ouhaibi Djellouli, Ahlem Djelti Messal, Meriem Abdi, Meriem Samia Aberkane, Abdelillah Chiali, Amine Derdour, Aicha Idder, Faouzia Zemani -Fodil

**Affiliations:** ^1^Laboratoire de Génétique Moléculaire et Cellulaire (LGMC), Université des Sciences et de la Technologie d’Oran Mohamed BOUDIAF- USTO-MB, BP 1505, El M'naouer, 31000 Oran, Algérie; ^2^Agence Thématique de Recherche en Sciences de la Santé (ATRSS)– Oran, Algérie; ^3^Ecole Supérieure des Sciences Biologiques d’Oran (ESSBO); ^4^Clinique Chiali, Oran, Algérie; ^5^Laboratoire de Génétique Médicale Appliquée à l'Ophtalmologie, Clinique Hammou Boutlélis Oran, Algérie

**Keywords:** Case–Control Study, FOXP3 Gene, IL4 Gene, Keratoconus, Polymorphisms, Western Algeria

## Abstract

**Purpose:**

The aim of this case–control study was to determine the impact of environmental factors on the predisposition to develop keratoconus in a sample of Western Algerian population. Subsequently, we were interested in the implication of two single nucleotide polymorphisms (SNPs) *IL4* rs2070874 and *FOXP3* rs3761548, previously described as contributing to the occurrence of allergy, in the development of keratoconus.

**Methods:**

The study included 70 unrelated KC cases and 70 controls originating from Western Algeria. DNA genotyping was done using predesigned probe-based allelic discrimination TaqManⓇ assays. Allele and genotype frequencies were compared between the cases and controls by Chi-square test and odds ratios with 95% confidence intervals.

**Results:**

A significant association between risk factors such as family history, atopy, eye rubbing, and the development of keratoconus was found in our sample. Smoking would provide a protective effect against the pathology. No statistically significant differences were found in the allele and genotype frequencies between cases and controls neither for *IL4* rs2070874 nor for *FOXP3* rs3761548.

**Conclusion:**

Our study provides, for the first time, a clear demonstration of the absence of association of the allergy-associated *IL4* and *FOXP3* polymorphisms with KC in a sample from Western Algerian population.

##  INTRODUCTION

Keratoconus (KC) is a degenerative bilateral corneal dystrophy characterized by gradual thinning of the cornea leading to a loss of visual acuity. KC is classified as a noninflammatory disease;^[1]^ however, several studies rejected this theory after the discovery of the expression of inflammatory mediators such as cytokines in the tears of patients suffering from KC.^[2,3]^

KC is a multifactorial disease resulting from the interaction of environmental and genetic factors. Although the etiology of KC is not clearly established, genetic and environmental factors such as allergy or eye rubbing seem necessary for disease expression,^[4]^ despite the positive associations found between atopy (allergy, asthma, eczema) and KC.^[5,6,7]^ No study has focused on the association between KC and polymorphisms in inflammatory mediators genes of these immune disorders.

Interleukin 4 is a pleiotropic cytokine produced by activated T-lymphocyte and mast cells.^[8]^ This cytokine plays a major role in type 2 immune responses characterized by the production of immunoglobulin E (IgE) and immunoglobulin G1 (IgG1).^[9]^ IgE is strongly implicated in atopic and allergic diseases.^[10]^ Several genetic variants in the *IL4* gene and its receptor *IL4*-R have been found associated with allergic rhinitis (AR).^[11]^ The *IL4* rs2070874 has been reported as associated with asthma and atopy in several studies.^[12,13]^ Hai-Jun Yang performed a meta-analysis and found that this polymorphism is correlated with increased asthmatic risk.^[14]^

The Forkhead box transcription factor (*FOXP3*) has an important role in the development and function of regulatory T cells (Tregs), and also in peripheral tolerance.^[15]^ Many severe lymphoproliferative diseases occur due to Treg deficiencies resulting from *FOXP3* mutation. Thus, Tregs mediate dominant tolerance to self and have also been shown to be equally important in the control of autoimmune diseases, allergy, fetal–maternal tolerance, allograft tolerance, and immunopathology.^[16]^ The rs3761548 polymorphism of *FOXP3* gene was identified as being associated with AR in heterozygous form in Han Chinese patients,^[17]^ and in Hungarian AR patients.^[18]^

To the best of our knowledge, this is the first study dedicated to the study of KC in Algeria and Africa in order to find a possible association between the inflammatory gene polymorphisms (rs2070874 and rs3761548) and the disease pathology.

##  METHODS

### Subjects

In total, 70 KC cases and 70 healthy controls originating from Western Algeria were included in this study. Patients were recruited from the specialized hospital, Institute of Ophthalmology EHS (établissemnt hospitalier specialisé) Hammou Boutelilis in Oran Algeria as well as from a private ophthalmology clinic. The control group included voluntary donors from the blood transfusion center of Oran hospital “CHUO” (Centre Hospitalo-Universitaire d'Oran), as well as student volunteers [Table 1].

**Table 1:** Study population's characteristics


**Characteristics**	**KC cases (%)**	**Healthy controls (%)**	***P*** **-value**
Number of subjects	70	70	–
Men	21 (30%)	40 (57%)	0.001
Women	49 (70%)	30 (43%)	
Age (mean ± SE), years	30.40 ± 11.33	30.14 ± 9.29	0.396
Family history of KC	15 (21%)	2 (3%)	0.0007
Consanguinity	21 (30%)	15 (21%)	0.24
Atopy	48 (69%)	11 (16%)	1 × 10 ${}^{-6}$
Eye rubbing	56 (80%)	1 (1%)	1 × 10 ${}^{-6}$
UV exposure	40 (57%)	50 (71%)	0.077
Exposure to cigarette smoke	27 (39%)	59 (84%)	1 × 10 ${}^{-6}$
KC, keratoconus; *P*, significance The values are presented as the mean ± standard error; the *P*-value for each comparison is calculated and is timed significant if it is superior than 0.05.			

**Table 2:** Details of the SNP used in the study


**Gene**	**db SNP ID**	**Assay ID**	**Location**	**Gene/Function**
*IL4 *	rs2070874	C__16176215_10	Chr.5: 132674018	5 Prime untranslated variant
*FOXP3 *	rs3761548	C__27476877_10	Chr.X: 49261784	Intron variant
SNP, single nucleotide polymorphism; db SNP, single nucleotide polymorphism database; Chr, chromosome				

**Table 3:** Genotypes and alleles distribution of *IL4* rs2070874 and *FOXP3* rs3761548 variants between keratoconus patients and control group


**Gene/SNP**	**Genotype/Allele**	**KC Patients ** ***n*** ** = 70 (%)**	**Controls ** ***n*** ** = 70 (%)**	**X²**	***P*** **-value**	**OR [CI]**
*IL4 *rs2070874	TT CC TC	6 (9) 43 (61) 21 (30)	3 (4) 49 (70) 18 (26)	1.62	0.44	_
	T C	33 (24) 107 (76)	24 (17) 116 (83)	1.78	0.18	0.67 [0.37–1.21]
	TT+TC CC	27 (39) 43 (61)	21 (30) 49 (70)	1.14	0.28	1.45 [0.72–2.91]
**Gene/SNP**	**Genotype/Allele**	**KC Patients ** ***n*** ** = 70 (%)**	**Controls ** ***n*** ** = 70 (%)**	**X²**	***P*** **-value**	**OR [CI]**
*FOXP3 *rs3761548	TT GG GT	12 (17) 32 (46) 26 (37)	12 (17) 33 (47) 25 (36)	0.03	0.98	_
	T G	50 (36) 90 (64)	49 (35) 91 (65)	0.02	0.900	1.03 [0.63–1.68]
	TT + GT GG	38 (54) 32 (46)	37 (53) 33 (47)	0.03	0.86	1.06 [0.55–2.05]
KC, keratoconus; P, significance; OR, odds ratio; CI, 95% confidence interval					

The present study was conducted according to the principles set out in the Declaration of Helsinki,^[19]^ and was approved by the ethics committee at the National Evaluation and Planning Committee of the Algerian University Research. An informed written consent was obtained from all participants.

Detection of KC was performed by an experienced ophthalmologist based on visual acuity assessment, findings of slit lamp as: corneal thinning, Vogt's striae,^[20]^ and Fleischer's ring and topographic data. Patients previously grafted for one or both eyes were considered as cases.

All KC patients with AR reported having common classical symptoms mentioned in Allergic Rhinitis and its Impact on Asthma (ARIA) guidelines-2016 revision,^[21]^ as nasal itching, sneezing, rhinorrhea, and nasal congestion, and other KC patients stated that they already suffered from ocular symptoms as allergic rhinoconjunctivitis associated with itching and redness of the eyes and tearing.

The 70 control subjects had no ocular disease or previous eye surgeries and no family ocular history; all controls wearing glasses or suffering from myopia were excluded.

### DNA Extraction

Eight mL peripheral blood was collected from patients and healthy controls using EDTA containing tubes and stored at –20°C until analyses. Genomic DNA was extracted using the salting out method.^[22]^

### Genotyping of *IL4* rs2070874 and *FOXP3* rs3761548 Polymorphisms 

Molecular genotyping of SNPs was performed using TaqManⓇ SNP genotyping assay (Applied Biosystems Foster City, CA, USA) [Table 2] on qTOWER³ real-time polymerase chain reaction (PCR) machine (Analytik Jena, Germany). A 20 µL PCR reaction contained 1X TaqManⓇ genotyping master mix (Applied Biosystems Foster City, CA, USA), 1X SNP genotyping assay mix, and 20 ng DNA. PCR cycling parameters included pre-denaturation at 60°C for 30 sec, denaturation at 95°C for 10 sec, followed by 50 cycles of denaturation at 95°C for 15 sec each, and finally annealing/extension at 60°C for 90 sec. The PCR products were measured at 60°C for 30 sec, which are proportional to the level of the fluorescence VICⓇ and FAMⓇ.

### Statistical Analysis

The Hardy–Weinberg equilibrium (HWE) in the control group was calculated using Chi-square test. The comparison of quantitative variants between KC patients and control group, the frequencies of the allele and genotype were analyzed using the Pearson's Chi-square (χ ${}^{2}$ ) test. *P*-values < 0.05 were considered significant. Risk was assessed by the odds ratios (ORs) and 95% confidence intervals (CIs) were also estimated.

The genotype relative risk (GRR) method (a single genotype versus the others) was calculated to compare the genotype distribution in patients and controls. All the analyses were done using 2 × 2 contingency tables.

##  RESULTS

The demographic and other characteristics of all subjects are shown in Table 1. We first looked at the sex ratio; the gender distribution between patients and controls was found to be significantly different (*P *= 1 × 10 ${}^{-3}$ ). The mean age of KC patients was 30.40 ± 11.33 (range 6–70 years), while the mean age of the healthy group was 30.14 ± 9.29 (range, 19–62 years), there was no statically significant difference in the mean age between the two groups. Then, we were interested in the most discriminating risk factors; we observed a clear significant difference between cases and controls concerning atopy, eye rubbing with a high number of cases than controls also exposure to cigarette smoke as risk factor but with a large number of controls than cases (*P *= 1 × 10 ${}^{-6}$ ). KC patients had more family history of the pathology than healthy controls (21% vs 3%, *P* = 7 × 10 *${}^{-4}$ *). We also noted that there was no statistically significant difference between the case and control groups regarding the consanguinity and ultraviolet radiation (UV) exposure (*P* = 0.24 and 0.077, respectively).

The distribution of allele frequencies for the two SNPs in control group did not deviate significantly from HWE (*P* > 0.05). The details of gentotypic and allelic frequencies are summarized in Table 3. The frequency distributions of *IL4* rs2070874 genotypes between cases and controls revealed no statistical significance (*P *= 0.44). The allelic distribution of the polymorphism in the control population revealed that the allele C of *IL4* rs2070874 represents the major allele (0.83) and the allele T represents the allele minor (0.17), there were no significant differences in the frequency's distribution of the *IL4* rs2070874 C/T alleles (*P =* 0.18) even in the presence of at least one copy of the allele T of *IL4* rs2070874 among the genotypes (*P =* 0.28).

The distributions of genotype's frequencies of *FOXP3* rs3761548 in KC cases were: GG, 46%; GT, 37%; and TT, 17%; and the distribution in control group were: GG, 47%; GT, 36%; and TT, 17%. No significant association was observed between KC patients and healthy controls concerning *FOXP3* rs3761548 genotypes (*P *= 0.98), the allele frequency distribution was also nonsignificant (*P *= 0.9; OR = 1.03; 95%CI [0.63–1.68]), with minor T allele frequency 0.35 and major G allele frequency 0.65. The presence of at least one copy of the allele T of *FOXP3* rs3761548 showed nonsignificant association between cases and controls (*P *= 0.86; OR = 1.06; 95%CI [0.55–2.05]).

##  DISCUSSION

We investigated the impacts of the most discriminating risk factors and *IL4* rs2070874 and *FOXP3* rs3761548 polymorphisms on a group of KC patients and controls from Western Algeria. To the best of our knowledge, this is the first study on the impact of *IL4* and *FOXP3* polymorphisms in a population originating from Algeria.

Our results showed a higher female prevalence in our study population (*P *= 0.001). This female predominance of KC found in our sample is the opposite of what is found in the literature. Some studies have shown a male predominance among patients from the United States,^[23]^ while others have shown no significant difference.^[24]^ These results remain to be confirmed on a larger number of patients in order to be able to explain this female predominance in our population [Table 1].

The mean age in our sample of patients is equivalent to that found in several studies that demonstrate that KC usually affects adolescents at puberty as well as young adults until the fourth decade of life.^[25]^ The mean age of KC cases in our study was 30.40 ± 11.33 years [Table 1], which fits perfectly with the study of Cozma et al^[26]^ concerning the White patient group in his work about the influence of ethnic origin on the incidence of KC.

In our sample, there was a significant difference between cases and controls groups concerning family history with KC (*P* = 7 × 10 ${}^{-4}$ ) [Table 1]. Indeed, the familial link with KC represents a serious risk factor,^[27]^ since Gordon-Shaag et al showed that the risk of developing KC was 15 to 67 times higher compared to the general population.^[28]^ In addition, they reported that 5 to 27.9% of patients with KC had a positive family history.^[28]^

Consanguinity has also been proposed as a risk factor of KC in Israeli Arabs and Palestinian Arabs,^[28,29,30]^ as studies have shown that the high number of consanguineous marriages among Muslims is a cause of the increased incidence of KC.^[31]^ However, we did not find any significant difference between cases and controls in our population for this factor (*P *= 0.24) [Table 1]; this could be caused by the small number of KC patients group.

For more than 50 years, atopy has been identified as a risk factor.^[32]^ In addition, several studies have shown that asthma and allergy are associated with KC with statistical significance (*P = *0.0008 for asthma and 0.04 for allergy).^[7]^ Our results correlate with this work since in our sample, atopy was reported in 69% of cases compared to 16% in controls with a statistically significant difference (*P *= 1 × 10 ${}^{-6}$ ) [Table 1], knowing that all patients are from Western Algeria – a coastal region known for its humid and marine climate and neighboring industrial areas. A study in the region of Eastern Algeria confirmed that climatology would increase the risk of developing allergic diseases, and atopy based on family criteria was very common in this population.^[33]^

Another risk factor is related to patients' habits, the eye rubbing. The rubbing of the eyes is currently the most important risk factor that is clearly identified. This risk factor triggers the onset and progression of the disease through several effects, including the stimulation of inflammation. Most authors report that approximately half of the KC patients rub their eyes, although this varies with the usual duration of rubbing and that the rubbing of the eyes is mild or vigorous.^[28,34]^ The results of our study are in perfect correlation with the literature. We have shown that 80% of patients with KC rubbed their eyes regularly compared to 1% in controls. Thus, in our sample, eye rubbing seems to be associated with the appearance of the KC (*P* = 1 × 10 ${}^{-6}$ ) [Table 1]. Indeed, several studies have attempted to explain the involvement of this risk factor in the development of KC. In fact, the micro-trauma caused to the epithelium by the eye rubbing generates high levels of matrix metalloproteases (MMP-1 and MMP-13) secreted by the epithelial and stromal cells, in addition, the release of inflammatory mediators (IL-6 and TNF-α) is one of the processes leading to KC and its progression.^[35]^

In our sample, we found that there was no statistically significant difference between the case and control groups concerning sun exposure risk factor (*P* = 0.07) [Table 1]. Studies have shown that the prevalence of KC is higher in sunny countries than in Europe or North America, high sun exposure in these countries would explain the high prevalence of KC.^[27]^ In addition to the genetic component, UV light has an additive effect by causing oxidative damage which would be among the causes of the acceleration of the pathological process of KC.^[28]^ However, it should be noted that UV could provide, at a controlled dose, a beneficial effect by inducing the reticulation of corneal collagen, thus attenuating the development or progression of the disease.^[36]^

In 2008, a study was able to demonstrate for the first time that cigarette smoking appeared to have a protective effect against KC.^[37]^ Our results seem to support this hypothesis, since in our sample, we observed a greater frequency of smoking in controls (84%) compared to KC patients (39%). Moreover, this difference is statistically significant (*P = *1 × 10 ${}^{-6}$ ) [Table I], and exposure to cigarette smoke appears to have a protective effect (OR = 0.1 [0.055–0.269]). Other studies concluded that there was no significant association between smoking and KC.^[30]^

The hypothesis of testing the effect of the *IL4* rs2070874 and *FOXP3* rs3761548 polymorphisms in the predisposition to KC development is based on the fact that these polymorphisms known to be associated with allergies could be either directly associated with the development of KC or indirectly, via inflammation induced by micro-trauma due to eye rubbing.^[34]^ Indeed, our results [Table 1] showed that atopy and eye rubbing in patients with KC were significantly associated with the pathology in our study sample (*P* *= *1 × 10 ${}^{-6}$ ) [Table 1].

According to our results, the T allele of *IL4* rs2070874 represents the minor allele (0.17) [Table 3]. This allelic distribution is close to the findings of a study performed in 2018 on a sample of 58 subjects in the Western Algeria (0.10).^[38]^ The same observation was reported in other populations such as Italians (0.12),^[39]^ Swedish, Australian, Finnish, and English populations (0.16).^[40]^ This frequency is lower than that reported in the African-Americans (0.43)^[41]^ and Indians (0.39).^[42]^ However, the T allele of *FOXP3* rs3761548 represents the minor allele with a frequency of 0.35 [Table 3]. This minor allele frequency (MAF) is in line with the Egyptian study (0.34),^[43]^ but different from those reported in Turkish and Indian populations, 0.61 and 0.56, respectively.^[44,45]^ This variability in the results of the studies may be due to ethnic factors and the size of the sample studied [Table 3].

Our case–control study suggests no association between (*IL4* rs2070874, *FOXP3* rs3761548) polymorphisms and susceptibility to KC, even in the presence of at least one copy of the allele T of *IL4* rs2070874 or T allele of *FOXP3* rs3761548 [Table 3]. According to Bawazeer et al,^[34]^ allergy is indirectly associated with the development of KC and its effect is probably due to inflammation caused by rubbing eye movements. These results, once confirmed in a larger cohort, would be further evidence.^[34]^ Indeed, most authors report that about half of the patients with KC rub their eyes regularly, although the percentage varies among studies.^[28]^ It is interesting to note that in cases of asymmetric KC, the most affected eye has been rubbed the most vigorously.^[46]^ Coyle et al reported the case of an 11-year-old boy who at the age of 5, discovered that he could stop his tachycardia by vigorously massaging his left eye (up to 20 min/day).^[47]^ At age 7, he hadn't developed anything in his eyes. At the age of 11, the child developed unilateral KC in only his left eye which was massaged vigorously. Another case of KC has been reported in a patient with a history of vigorous daily massage of the left eye which led to unilateral KC in that eye only.^[48]^ A series of cases confirms the asymmetrical expression of the disease in patients who usually rub the most affected eye.^[46,49,50]^

Thus, eye rubbing would cause micro-trauma leading to the secretion of high levels of MMP.^[35]^ These factors can lead to progression of KC associated with apoptosis of keratocytes,^[51]^ and atopic episodes can contribute and interact with other inflammatory processes related to KC.^[52]^

This is the first demonstration of the implication of susceptibility genes to allergy in the development of KC, and this is the first time that the *IL4* and *FOXP3* genes association was analyzed with KC risk in an Algerian population.

Our results showed that there would be a significant association in our study sample between risk factors such as family history with disease, atopy and eye rubbing, and KC development. In addition, our results confirmed that smoking would provide a protective effect against the pathology. However, it has been shown that there is no association between the *IL4* rs2070874 and *FOXP3* rs3761548 polymorphisms and the occurrence of KC in the population of Western Algeria. However, because of the moderate size of the sample, the statistical power of the present study was relatively low, being 50% and 59% for the *IL4* rs2070874 and *FOXP3* rs3761548 polymorphisms, respectively.

In summary, our results provide precise and up-to-date information on the association of the two polymorphisms studied with KC in the population of Western Algeria. The small size of our study requires a replication of the results in a larger sample of the population in order to confirm or affirm the association. Nevertheless, the present study does not exclude the effect of *IL4* and *FOXP3* gene polymorphisms in the pathophysiological process of the KC disease. For this reason, the present study may be considered as a pilot study.

##  Financial Support and Sponsorship

Nil.

##  Conflicts of Interest 

There are no conflicts of interest.
